# Retraction: A network map of cytoskeleton‐associated protein 4 (CKAP4) mediated signaling pathway in cancer

**DOI:** 10.1002/ccs3.12043

**Published:** 2024-07-11

Retraction: Suchitha, G. P., Balaya, R. D. A, Raju, R., Prasad, T. S. K, Dagamajalu, S. (2023) A network map of cytoskeleton‐associated protein 4 (CKAP4) mediated signaling pathway in cancer. *Journal of Cell Communication and Signaling*, *17*: 1097–1104. https://doi.org/10.1007/s12079‐023‐00739‐w.

The above article, published online on March 21, 2023 in Wiley Online Library (wileyonlinelibrary.com), has been retracted by agreement between the authors, journal Editor‐in‐Chief Bernard Perbal and John Wiley & Sons, Ltd. The retraction has been agreed following a report by a third party which described methodological errors in the published article. The authors have confirmed that the article contains annotation errors caused by the same alternate names for two different proteins. The editor and authors agree that the conclusions are fundamentally impacted by this error and that the article must be retracted.

